# Medication reconciliation at admission and discharge: a time and motion study

**DOI:** 10.1186/1472-6963-13-485

**Published:** 2013-11-21

**Authors:** Ari N Meguerditchian, Stanimira Krotneva, Kristen Reidel, Allen Huang, Robyn Tamblyn

**Affiliations:** 1 Clinical and Health Informatics Research Group, McGill University, Montreal, QC H3A 0G4, Canada; 2 Department of Surgery, McGill University, Montreal, QC H3A 0G4, Canada; 3 Department of Oncology, McGill University, Montreal, QC H3A 0G4, Canada; 4 Division of Geriatric Medicine, Department of Medicine, University of Ottawa & the Ottawa Hospital, Ottawa, ON K1H 8L6, Canada; 5 Department of Epidemiology, Biostatistics and Occupational Health, McGill University, Montreal, QC H3A 0G4, Canada; 6 Department of Medicine, McGill University, Montreal, QC H3A 0G4, Canada

**Keywords:** Medication reconciliation, Admission, Discharge, Adverse drug events, Prescription, Electronic records

## Abstract

**Background:**

Medication reconciliation at admission, transfer and discharge has been designated as a required hospital practice to reduce adverse drug events. However, implementation challenges have resulted in poor hospital adherence. The aim of this study was to assess the processes required to carry out medication reconciliation: the health professionals involved, the tasks and time devoted to medication reconciliation in general hospital settings.

**Methods:**

A time-and-motion study design was used. Using a systematic sample of patients admitted and discharged from geriatric, medical and surgical units in two academic centers, health professionals involved in medication reconciliation were observed and timed. Descriptive statistics were used to summarize the number of professionals involved, tasks performed, and mean time devoted.

**Results:**

Up to 3 professionals from 2 disciplines (medicine and pharmacy) were involved in the medication reconciliation process. Geriatric reconciliations took the most time to complete at admission (mean: 92.2 minutes (SD = 44.3)) and discharge (mean: 29.0 minutes (SD = 23.8)), followed by internal medicine at admission (mean: 46.2 minutes (SD = 21.1)) and 19.4 (SD = 11.7) minutes at discharge) and general surgery minutes at discharge (mean: 9.9 minutes (SD = 18.2)). Considerable differences in order, type and number of tasks performed were noted between and within units. Tasks independent of direct patient interaction took more than twice the time required to complete than tasks requiring patient interaction.

**Conclusion:**

Lack of coordination, specialized training and agreement on the roles and responsibilities of professionals are among the most probable reasons for work-flow inefficiencies, possibly variability in quality, and time required for the current medication reconciliation process. A better understanding of the admission processes in general surgery is required. Standardization and use of electronic tools could improve efficiency and hospital adherence.

## Background

Adverse drug events (ADEs) are the sixth leading cause of death in the United States [1] and represent a significant financial burden to health care institutions at an estimated cost of $5.6 million per hospital per year [2]. Approximately one-quarter of patients experience an ADE after hospital discharge [3]. At least 58% of these ADEs are preventable, as they result from incomplete drug information received by hospitals, prescribing or dispensing errors, and overuse or underuse of medications [4]. In fact, at admission, 60-70% of medication histories contain at least one error such as omitting a certain medication [5-7]. The most commonly omitted medications are cardiovascular drugs, pain medications, anti-infectious medications, and central nervous system medications, such as anti-depressants and sleeping pills [8]. Failure to obtain a complete and accurate pre-admission medication history is also responsible for most ADEs after discharge [4,9].

Medication reconciliation has been recommended to reduce adverse drug events. Defined as a “formal process of obtaining a complete and accurate list of each patient’s current home medications – including name, dosage, frequency and route – and comparing the physician’s admission, transfer, and discharge orders to that list” [10], medication reconciliation has been associated with reductions in discrepancy rates [11-14]. Based on evidence for its positive impact, medication reconciliation has recently been designated as a required organizational practice by hospital accreditation authorities in Canada [15] and the United States [16]. In compliance with accreditation standards, most hospitals have instituted some form of the medication reconciliationreconciliation process; however, hospital adherence is poor, with less than 20% of patients at-risk of ADEs receiving a complete medication history review [17-20].

Although nurses and physicians believe that medication reconciliation is associated with significant improvements in patient safety [11], resource-intensiveness cited as the main reasons for the current low rates of adherence [8,17,18,21-27]. There is limited empirical information about the process and time required by various health care professionals to implement medication reconciliation [28]. The availability of such information could allow for the development of more efficient planning and allocation of resources to medication reconciliation protocols, which could ultimately improve hospital adherence. The purpose of this study was to assess the processes required to carry out medication reconciliation in the geriatrics, internal medicine and general surgery units of two Canadian Hospitals by observing the health professionals involved, tasks and time devoted

## Methods

### Setting

This study took place between January 1^st^ and August 31^st^, 2011 at two urban, tertiary care academic hospitals affiliated with the McGill University Health Centre (MUHC) in Montreal, Canada: the Royal Victoria Hospital (517 beds and approximately 22,000 admissions per year) and the Montreal General Hospital (417 beds and approximately 15,000 admissions per year) [29]. Ethical approval for the study was obtained from the Research Ethics Office at the MUHC.

### Design and study population

A time and motion study [30] design was used to assess the processes and time required for medication reconciliation in three hospital units: geriatrics, medicine and surgery. These units were selected for their clinical significance in treating patients at high-risk of ADEs due to case complexity and vulnerability to medication errors. In each unit, we selected the first 10 admissions and first 10 discharges for assessment who: (1) had active prescriptions prior to hospitalization and (2) came from, or were being discharged, home. This sample size provides a 95% confidence interval width of approximately 13 minutes per reconciliation assuming a standard deviation of 15 minutes [31,32]. Admission medication reconciliations on the general surgery unit were excluded due to challenges, specific to this unit. Finally, we excluded patients without active prior prescriptions, or patients coming from, or being discharged to another acute or chronic care facility, as these patients also differed too much from the typical acute care cases.

### Tasks and timing of medication reconciliation processes

To determine the tasks performed during a typical medication reconciliation, interviews were conducted with health professionals known to be involved in the process in the units of geriatrics (N = 4) and internal medicine (N = 2). The following tasks were defined for medication reconciliation at admission: (1) tasks completed *prior to meeting the patient*, (2) tasks completed while in *discussion with the patient*, (3) tasks involving *external research on the medication history*, and (4) tasks related to the *reconciliation of medications and documentation* of the entire process, including recommendations for in-hospital prescriptions. Only one major task, *preparing the discharge prescription*, was selected for timing during medication reconciliation at discharge. A detailed description of what actions were timed for each task is presented in Table 1.

**Table 1:** Description of tasks and subtasks involved in admission or discharge medication reconciliation

**Task/Subtask**	**Description**
**Admission: *prior to meeting the patient***	
Reading the medical chart	Reviewing patients’ charts for demographic information, reading the admission note, reading admission orders, reviewing allergies and other health or medication related information that may have been collected at the emergency room or in prior, recent admissions, and taking notes.
Reviewing lab results	Reviewing patients’ lab results and writing notes.
Calculations	Calculating and recording creatinine clearance, phenytoin, and other clinical values.
**Admission: *discussion with patient***	
Introduction	Greeting patients or family members. It also may include asking patients who gets their medication from the pharmacy.
Community drug list	Interviewing the patient about his or her prescription medications at home.
Medication knowledge	Interviewing patients to determine if they know why they are taking the drugs they have prescriptions for (therapeutic intention). Most of the time this question is not asked directly or the patient initiates this discussion on his or her own.
Medication posology	Interviewing patients about the frequency at which they take their drugs.
Adherence to medication	Asking patients if they actively take their medications as prescribed and if not, why not.
Over-the-counter medication	Asking patients if they take any over the counter medications such as acetaminophen, vitamins, or herbal remedies.
Allergies	Asking patients about any allergies they may have to medication.
Pharmacy coordinates	Asking patients for the phone number or exact address of their community pharmacy.
Permission to exchange medication information	Informing patients that their pharmacy will be contacted in order to get information on their community medications. Sometimes the permission is implied by providing the pharmacy’s coordinates, and is therefore timed under pharmacy coordinates, but often clinicians directly ask the patient if they allow them to call their pharmacy.
**Admission: *external research on medication history***	
Phoning the patient's family member or caregiver	Communicating with the patient’s family member or caregiver by phone.
Phoning the patient's CLSC/nursing home	Communicating with the patient's CLSC or nursing home by phone.
Searching for pharmacy coordinates	Performing a Google search on the internet to find out patient's pharmacy's phone number. This task also includes time searching for the patient's CLSC phone number, if applicable.
Communicating with the pharmacy	Communicating with the pharmacy by phone with the purpose of obtaining a fax of the patient’s medication profile, validating their compliance, determining how they manage their medications (using a pillbox or other methods), and asking about recent medication changes.
Waiting for pharmacy fax	Measures the wait-time between calling the patient's community pharmacy to ask for a fax of their medication list, and actually receiving it. While waiting, the pharmacist either does other work unrelated to this patient, or they do other tasks for this admission such as reading the patient's lab results (in which case, the time would also be recorded under the appropriate task heading).
**Admission: *reconciliation of medications and documentation***	
Clarifying discrepancies with patient	Discussing with patients any discrepancies between what the pharmacy’s community drug list, and the list of medications that patients had verbally provided.
Documenting/Reviewing admission medication list	Documenting the community drug list and reviewing in hospital medications that were given to the patient at admission. This task also includes time spent looking at vital signs from the patient’s clipboard, if this occurs.
Discussing recommendations with in-hospital prescriber	Discussing recommendations by the pharmacist/pharmacy student for the patient's in-hospital medications with the physician or resident.
Documenting recommendations for in-hospital prescriber	Documenting recommendations regarding the patients’ in-hospital medications in the chart progress notes.
Other documentation	Timing any other notes the clinician records that do not fall in the above categories but that are related to the patients’ medications.
**Discharge: *preparing the discharge prescription***	
Reviewing in-hospital medications and labs	Reviewing in-hospital medication lists and labs, in any of the following locations: paper medical chart, nurse’s kardex, hospital pharmacy database, or electronic hospital medical record.
Reviewing community medications or chart notes	Reviewing available documentation on patient's community medications or any other chart notes.
Discussing in-hospital medications with patient	Discussing in-hospital medications with the patient.
Discussing community medications with patient	Discussing medications the patient was taking in the community prior to his or her hospital admission with the patient.
Writing the discharge prescription	Writing or reviewing the discharge prescription, if both were done at the same time. Other actions timed under this heading included: communicating with other health professionals regarding questions about the patient or his or her medications, using online programs to research drugs, or searching for allergies in the patient's chart.
Reviewing the discharge prescription	Reviewing the prescription by the prescribing physician, resident or pharmacist before signing it. When the discharge prescription was written by a physician or resident, this task included time spent by the pharmacist reviewing it before the physician or resident signed it.
Discussing the discharge prescription with the patient	Discussing the discharge prescription with the patient and/or family/caregiver. It may also include documenting explanations about the discharge prescription for the patient.
Consulting with other clinicians about the discharge prescription	Asking other collaborating health professionals questions regarding the patient’s discharge prescription (in person, or by phone).
Revising the discharge prescription after consulting other clinicians	Editing the discharge prescription after having consulted other clinicians about it.
Revising the discharge prescription after consulting the patient	Editing the discharge prescription after having discussed it with the patient.
Other final documentation	Preparing any other documentation regarding the patient’s medication plan such as documentation that may be needed by the community pharmacist.
Faxing discharge information to community pharmacy	Faxing a summary of medication changes or the discharge prescription to the community pharmacy.
Self-reported time	Any additional time spent on discharge medication reconciliation by the health professional before the research assistant arrived to start timing. For example, in some cases the pharmacist had started looking at the patient's chart before the research assistant had arrived to start timing.

Data was collected by two trained research assistants using portable laptops. This allowed the research assistants to follow the patient when visits to different health professionals were required to complete the medication reconciliation process. Health care professionals, known to be involved the process, were approached, if they were on duty at the time of the study, and asked for their consent to be timed by a research assistant (N = 53). Health professionals were shadowed on the units. Timing software was created in Microsoft Access 2003 (Microsoft Corporation, Redmond, WA) to collect information on the time required for each task. The software was designed to allow a trained research assistant to click once beside a task from the user’s list in order to start timing, and then to click again in order to stop the timing. For each pair of clicks the following information was logged in the database: research assistant’s initials, name and type of health care professional, task being timed, and an anonymous patient identification. No patient information such as clinical or demographic characteristics was recorded. If multiple health care professionals were involved in performing the medication reconciliation tasks for the same patient, each professional was timed for their respective contribution. Data collection was done between Monday and Friday, from 7:00 AM to 6:00 PM. We specifically limited our study to these time periods to characterize the medication reconciliation process during optimal staffing conditions.

### Statistical analysis

Descriptive statistics were produced, summarizing the number and types of health care professionals involved as well as the tasks and time devoted to medication reconciliation at admission and discharge in each unit. To determine the number and types of different health care professionals involved, the health care professionals who participated in one or more tasks were counted per medication reconciliation, separately for admission and discharge. The frequency of having one, two or three health care professionals involved in the reconciliations was then calculated by type of health care professional and by unit. To determine the mean overall time devoted per type of reconciliation (admission or discharge), the total time spent by health care professionals was first added per task, then for all tasks, and then divided by the total number of reconciliations in that unit. All analyses were performed using SAS software (SAS version 9.2, Institute, Inc., Cary, North Carolina).

## Results

### Health care professionals involved in medication reconciliation

All of the approached health care professionals (N = 40) provided written consent to have their work observed and timed by a research assistant. These professionals consisted of 11 pharmacists, 4 pharmacy students, 7 physicians, 12 residents, and 6 medical students. In total, 103 medication reconciliations were observed: 41 at admission (21 from geriatrics and 20 from internal medicine) and 62 at discharge (21 from geriatrics, 21 from internal medicine and 20 from general surgery) (Table 2). The number and type of health care professionals who participated in the medication reconciliation process varied according to type (admission or discharge) and hospital unit. At admission, one health care professional (pharmacist or pharmacy student) was involved in 81.0% and 100.0% of observed reconciliations in geriatrics and internal medicine, respectively. At discharge, a single resident or medical student was involved in medication reconciliation in surgery, whereas two or three health professionals were involved in the majority of discharge reconciliations in geriatrics and internal medicine. The process involved one health care professional (pharmacist, physician or resident) in only 19.0% of observed reconciliations in geriatrics and internal medicine.

**Table 2:** Number and type of health care professionals participating in medication reconciliations per hospital unit

**Medication reconciliation type**^**a**^	**Admission**		**Discharge**		
	**Geriatrics (N = 21)**	**Internal medicine (N = 20)**	**Geriatrics (N = 21)**	**Internal medicine (N = 21)**	**General surgery (N = 20)**
	**N (%)**	**N (%)**	**N (%)**	**N (%)**	**N (%)**
**1 clinician involved**					
Total	17 (81.0)	20 (100.0)	4 (19.0)	4 (19.0)	20 (100.0)
Pharmacist	16 (76.2)	17 (85.0)	1 (4.8)	0 (0.0)	0 (0.0)
Pharmacy student	1 (4.8)	3 (15.0)	0 (0.0)	0 (0.0)	0 (0.0)
Resident	0 (0.0)	0 (0.0)	0 (0.0)	4 (19.1)	19 (95.0)
Medical student	0 (0.0)	0 (0.0)	0 (0.0)	0 (0.0)	1 (5.0)
Physician	0 (0.0)	0 (0.0)	3 (14.3)	0 (0.0)	0 (0.0)
**2 health care professionals involved**					
Total	4 (19.0)	0 (0.0)	16 (76.2)	14 (66.7)	**0 (0.0)**
Medical student and pharmacy student	0 (0.0)	0 (0.0)	0 (0.0)	1 (4.8)	0 (0.0)
Physician and pharmacy student	0 (0.0)	0 (0.0)	4 (19.0)	0 (0.0)	0 (0.0)
Pharmacist and pharmacy student	0 (0.0)	0 (0.0)	0 (0.0)	0 (0.0)	0 (0.0)
Pharmacist and medical student	4 (19.0)	0 (0.0)	0 (0.0)	0 (0.0)	0 (0.0)
Resident and medical student	0 (0.0)	0 (0.0)	0 (0.0)	1 (4.8)	0 (0.0)
Pharmacist and resident	0 (0.0)	0 (0.0)	1 (4.8)	2 (9.6)	0 (0.0)
Pharmacist and physician	0 (0.0)	0 (0.0)	11 (52.4)	10 (47.6)	0 (0.0)
**3 health care professionals involved**					
Total	0 (0.0)	0 (0.0)	1 (4.8)	3 (14.3)	0 (0.0)
Pharmacist, resident and pharmacy student	0 (0.0)	0 (0.0)	0 (0.0)	1 (4.8)	0 (0.0)
Pharmacist, resident and medical student	0 (0.0)	0 (0.0)	0 (0.0)	1 (4.8)	0 (0.0)
Pharmacist and 2 residents	0 (0.0)	0 (0.0)	0 (0.0)	1 (4.8)	0 (0.0)
Pharmacist, physician and pharmacy student	0 (0.0)	0 (0.0)	1 (4.8)	0 (0.0)	0 (0.0)

*Abbreviations*: *Y* Year, *N* Number.

^a^Per type and number of health care professionals involved involved.

### Time required for completion of tasks related for admission medication reconciliation

Admission reconciliation took twice as long to conduct in geriatrics compared to internal medicine (mean 92.2 minutes (SD = 44.3) and 46.2 minutes (SD = 21.1)) (Table 3). Similarities between both units were also noted. Tasks independent of direct patient interaction, such as *reconciliation of medications and documentation* (40.8 minutes (SD = 20.0) and 18.9 minutes (SD = 15.0)) and tasks completed *prior to meeting the patient* (29.0 minutes (SD = 22.2) and 16.3 (SD = 9.3)) took more than twice the mean time it took to complete tasks that involved direct patient interaction such as *discussion with patient* (13.4 minutes (SD = 9.1) and 5.2 minutes (SD = 4.0) for geriatrics and internal medicine, respectively) and *external research on medication history* (13.0 minutes (SD = 11.9) and 6.2 minutes (SD = 4.2)). These four major tasks were most consistently done for admission reconciliations in both units (frequencies ranging from 90.5-100.0%).

**Table 3:** Time to complete medication reconciliation tasks at admission per hospital unit

**Medication reconciliation tasks**	**Geriatrics**				**Internal medicine**			
		**Time (minutes)**				**Time (minutes)**		
	**N (%)**	**Mean (SD)**	**Min**	**Max**	**N (%)**	**Mean (SD)**	**Min**	**Max**
**Overall**	21	92.2 (44.3)	46.1	202.5	20	46.2 (21.1)	22.5	94.6
***Prior to meeting the patient***	19 (90.5)	29 (22.2)	6.9	91.7	20 (100.0)	16.3 (9.3)	7.1	36.8
Reading medical chart	19 (90.5)	20.8 (18.6)	0.4	75.6	20 (100.0)	13.1 (8.1)	5.6	32.4
Reviewing lab results	19 (90.5)	7.8 (4.3)	3.4	21.3	20 (100.0)	3.2 (1.6)	1.0	7.0
Calculations	8 (38.1)	1 (0.6)	0.5	1.8	2 (10.0)	0.2 (0.0)	0.2	0.2
***Discussion with patient***^**a**^	21 (100.0)	13.4 (9.1)	0.6	31.0	20 (100.0)	5.2 (4.0)	1.1	15.5
Introduction	21 (100.0)	4 (3.3)	0.3	11.6	19 (95.0)	1.4 (2.1)	0.1	8.3
Community drug list	19 (90.5)	3.7 (3.3)	0.1	11.5	19 (95.0)	1.7 (1.8)	0.1	6.4
Medication knowledge	10 (47.6)	1.9 (1.5)	0.5	5.1	4 (20.0)	0.2 (0.3)	0.1	0.6
Medication posology	12 (57.1)	1.9 (1.6)	0.3	5.1	9 (45.0)	0.6 (0.4)	0.2	1.5
Adherence to medication	7 (33.3)	0.8 (0.4)	0.3	1.5	4 (20.0)	0.2 (0.2)	0.0	0.5
Over-the-counter medication	15 (71.4)	3.5 (2.5)	0.5	7.9	14 (70.0)	0.9 (0.9)	0.1	3.6
Allergies	14 (66.7)	0.9 (0.7)	0.2	2.9	11 (55.0)	0.2 (0.2)	0.1	0.7
Pharmacy coordinates	12 (57.1)	0.8 (0.5)	0.2	1.6	17 (85.0)	1.3 (2.7)	0.3	11.7
Permission to exchange medication information	7 (33.3)	0.7 (0.7)	0.1	1.8	9 (45.0)	0.2 (0.3)	0.0	1.0
***External research on medication history***^**b**^	19 (90.5)	13 (11.9)	4.0	47.1	19 (95.0)	6.2 (4.2)	1.0	19.2
Communication with family caregiver not in hospital	3 (14.3)	9.7 (4.8)	4.2	12.6	2 (10.0)	3.4 (3.4)	1.0	5.8
Communication with Community Service Centre/nursing home	1 (4.8)	6.4 (.)	6.4	6.4	1 (5.0)	2.1 (.)	2.1	2.1
Searching for pharmacy coordinates	11 (52.4)	5.1 (7.9)	0.5	28.3	11 (55.0)	3 (2.1)	0.4	5.5
Communication with the pharmacy	19 (90.5)	8.2 (9.4)	2.5	44.8	18 (90.0)	4.2 (3.4)	0.6	13.0
***Reconciliation of medications and documentation***	21 (100.0)	40.8 (20.0)	19.0	85.9	20 (100.0)	18.9 (15.0)	2.5	57.1
Clarifying discrepancies with patient	4 (19.0)	1.7 (1.2)	0.1	3.0	6 (30.0)	3.6 (3.2)	0.6	9.6
Documenting admission medication list	21 (100.0)	32.3 (17.8)	8.8	64.3	20 (100.0)	15.3 (11.7)	2.3	45.5
Discussing recommendations with in-hospital prescriber	8 (38.1)	3.1 (2.2)	0.6	7.3	4 (20.0)	2 (1.6)	0.7	4.3
Documenting recommendations for in-hospital prescriber	11 (52.4)	12.3 (10.3)	0.1	38.6	9 (45.0)	2.2 (1.8)	0.4	5.4
Other documentation	5 (23.8)	2 (1.0)	1.2	3.7	4 (20.0)	5.1 (5.0)	0.6	11.6

*Abbreviations*: *N* number, *SD* standard deviation, *Min* minimum, *Max* maximum.

^a^Includes any family members that may have been present.

^b^Includes a communication by phone, fax, or e-mail.

In both units, documenting and reviewing the admission medication list, a subtask of *reconciliation of medications and documentation*, required the most time compared to other subtasks (32.3 minutes (SD = 17.8) and 15.3 minutes (SD = 11.7)), followed by reading the medical chart, a subtask competed *prior to meeting the patient* (20.8 minutes (SD = 18.6) and 13.1 minutes (SD = 8.1)). Other time-consuming subtasks observed in both units included communication with the community pharmacy (8.2 minutes (SD = 9.4) in geriatrics and 4.2 (SD = 3.4) in internal medicine), and time spent on the phone with caregivers (9.7 minutes (SD = 4.8) in geriatrics and 3.4 minutes (SD = 3.4) in internal medicine). In addition, we observed considerable variability in the subtasks done in each unit. For example, the subtask of assessing patients’ medication knowledge was done in 47.6% of geriatric admission reconciliations, compared to only 20.0% of internal medicine ones. The order of performance of subtasks changed occasionally depending on the health professional performing the reconciliation.

### Time required for completion of tasks related to discharge medication reconciliation

Medication reconciliation at discharge took nearly half the time of admission reconciliation (Table 4). The process took the most time in geriatrics (mean time of 29.0 minutes, (SD = 23.8)), followed by internal medicine (19.4 minutes, (SD = 11.7)) and general surgery (9.9 minutes, (SD = 18.2)). There was significant variability between units in terms of the subtasks of *preparing the discharge prescription*. For example, the subtask of *writing the discharge prescription* was most consistently done across hospital units per reconciliation and took a mean time of 7.1 minutes (SD = 6.5) in geriatrics, 5.5 minutes (SD = 3.9) in internal medicine, and 3.4 minutes (SD = 2.6) in general surgery. Community medications were reviewed in 90.5%, 52.4% and 45.0% of observed discharge reconciliations and took a mean time of 1.9 minutes (SD = 1.0), 2.3 minutes (SD = 2.4), and 1.4 minutes (SD = 1.5) in geriatrics, internal medicine and general surgery, respectively. Prior to *preparing the discharge prescription*, clinicians rarely consulted their patients concerning their community medications (0%, 4.8% and 0% of reconciliations in geriatrics, internal medicine, and general surgery, respectively). Furthermore, the discharge prescription was rarely discussed with the patient (19%, 28.6% and 15% of reconciliations in geriatrics, internal medicine, and general surgery, respectively) or faxed to the patient’s community pharmacy (9.5%, 0% and 0% of reconciliations in geriatrics, internal medicine, and general surgery, respectively). Although not consistently performed within the units, the most time-consuming subtasks included: *preparing documentation* in addition to the discharge prescription in geriatrics (mean time of 29.8 minutes (SD = 12.7)), *writing the discharge prescription* in internal medicine (5.5 minutes (SD = 3.9)) and for three patients in surgery, *discussing the discharge prescription* (25.0 minutes (SD = 43.0)).

**Table 4:** Time to complete medication reconciliation tasks at discharge per hospital unit

**Medication reconciliation tasks**	**Geriatrics**				**Internal medicine**				**General surgery**			
		**Time (minutes)**				**Time (minutes)**				**Time (minutes)**		
	**N (%)**	**Mean (SD)**	**Min**	**Max**	**N (%)**	**Mean (SD)**	**Min**	**Max**	**N (%)**	**Mean (SD)**	**Min**	**Max**
*Preparing the discharge prescription*	21	29.0 (23.8)	5.2	91.7	21	19.4 (11.7)	1.2	44.0	20	9.9 (18.2)	1.9	84.0
Reviewing in-hospital medications	19 (90.5)	2.4 (1.5)	0.1	6.2	18 (85.7)	2.4 (2.2)	0.0	8.1	15 (75.0)	2.2 (2.0)	0.3	7.8
Reviewing community medications or chart notes	18 (85.7)	1.9 (1.0)	0.6	4.4	11 (52.4)	2.3 (2.4)	0.2	8.5	9 (45.0)	1.4 (1.5)	0.2	5.2
Self-reported time^a^	1 (4.8)	5 (.)	5.0	5.0	3 (14.3)	4.7 (3.5)	1.0	8.0	0 (0.0)			
Discussing community medications with patient	0 (0.0)				1 (4.8)	1.4 (.)	1.4	1.4	0 (0.0)			
Discussing the discharge prescription with patient	4 (19.0)	20.0 (13.4)	7.4	38.8	6 (28.6)	5.1 (2.7)	2.5	8.4	3 (15.0)	25 (43.0)	0.2	74.7
Consulting with other health professionals about the discharge prescription	14 (66.7)	5.3 (7.4)	0.0	27.7	13 (61.9)	5.3 (4.2)	0.1	15.5	5 (25.0)	1.8 (1.8)	0.2	4.2
Writing the discharge prescription	21 (100.0)	7.1 (6.5)	0.4	23.7	21 (100.0)	5.5 (3.9)	1.1	19.7	19 (95.0)	3.4 (2.6)	0.6	9.4
Reviewing the discharge prescription	17 (81.0)	4.7 (2.8)	1.0	11.4	16 (76.2)	4 (4.9)	0.1	19.9	2 (10.0)	0.7 (0.3)	0.6	0.9
Revising the discharge prescription after consulting other clinicians	7 (33.3)	1.6 (1.6)	0.0	4.8	3 (14.3)	1.3 (1.9)	0.1	3.5	0 (0.0)			
Revising the discharge prescription after consulting the patient	1 (4.8)	1.5 (.)	1.5	1.5	0 (0.0)				0 (0.0)			
Other final documentation	4 (19.0)	29.8 (12.7)	19.3	48.0	1 (4.8)	0.8 (.)	0.8	0.8	1 (5.0)	1.6 (.)	1.6	1.6
Faxing discharge information to pharmacy	2 (9.5)	3.8 (3.8)	1.1	6.5	4 (19.0)	9.9 (6.5)	2.3	16.2	0 (0.0)			

*Abbreviations*: *N* number, *SD* standard deviation, *Min* minimum, *Max* maximum.

^a^Any additional time spent by a health care professional looking at the patient's chart before a research assistant arrived to start timing.

## Discussion

In this study, we examined the workforce and time requirements to conduct medication reconciliation at admission and discharge in geriatrics, internal medicine and general surgery at two Canadian hospitals. First, up to three different health care professionals from two different disciplines were involved in the medication reconciliation process. Specifically, medication reconciliations in geriatrics and internal medicine most often involved one health care professional at admission (pharmacist or pharmacy student), and two at discharge (physicians and pharmacists), while in general surgery there was only one (usually junior) health care professional (resident or medical student). Second, a large number of tasks and subtasks were performed during the process; however there was large variation in their execution, both between and within study units. For example, the subtask of *assessing patients*’ *medication knowledge* was done in 47.6% of geriatric admission reconciliations, compared to only 20.0% of internal medicine reconciliations. An even greater difference in subtask performance between units was noted at discharge. In addition, the difference in time spent on medication reconciliation varied greatly between units, ranging from an average of 10 minutes (general surgery) to 92 minutes (geriatrics). Finally, the time spent on non-direct patient interaction tasks, such as *reconciliation* and *documentation*, *faxing discharge information to pharmacies*, *and consulting health care professionals*, was more than twice the time spent on tasks involving direct patient contact.

Our findings suggest workflow inefficiencies in medication reconciliation, which could be compromising the overall efficiency, quality, and institutional ability to implement the process. The number and types of health care professionals involved, as well as the inconsistencies of involvement within units, reflect a lack of consensus with regards to each profession’s roles and/or responsibilities, as well as a lack of specialized training for conducting medication reconciliation [33]. Recent studies have reported similar observations, indicating involvement of multiple professionals, including clinical pharmacists, physicians and students [8,33,34]. A third discipline, nursing, is also commonly cited as being involved in many settings [8,11,35,36]. The involvement of only junior staff in general surgery raises a concern regarding the quality of medication reconciliation and the competency of the staff. The observed variability of tasks, subtasks and their order of performance between and within units is also consistent with other studies [37,38], however, few studies compare the tasks and subtasks performed during medication reconciliation in the different hospital units.

While geriatric reconciliations were expected to be longer, due to the fact that the number of medications generally increases with age [39], the short length of discharge medication reconciliation in general surgery raises concerns regarding the ability to prevent post-discharge adverse events. In addition, no pharmacists were available in general surgery and recommended tasks, such as *discussing community medications* with patient and *faxing discharge information* to pharmacies, were rarely completed. We weren’t able to observe admission reconciliations in general surgery due to implementation challenges specific to this unit. These challenges were mainly caused by differences in patient flow: elective admissions, during regular business hours for planned surgical procedures, and urgent admissions for immediate acute care surgery, often outside of regular hours of operation. For elective surgeries, admission medication reconciliation was usually done by nurses in pre-operative clinics. For emergency surgeries, admitted through the emergency department, medication reconciliation was done on the way to, in the operating room, or never. Similar challenges in observing admission medication reconciliation in general surgery have been reported by a recent study, conducted in Ontario (Canada) [18]. Finally, there was a feeling of general discomfort among surgical staff, regarding the management of complex drug lists for conditions outside their areas of expertise.

In order to insure patient safety at low cost, optimizing medication reconciliation is essential. Despite increasing the demand for health professionals, involvement of more than one type of professional (particularly pharmacists and physicians) can enhance the quality of the overall process. For example, involvement of pharmacy staff during medication reconciliation results in obtaining an accurate medication history more often than when pharmacy staff are not involved [37]. However, clarification of the roles of each participant (including the patient and/or caregiver), taking into account the varying structures and resources at healthcare sites is a necessary step to avoid duplication and overlap, while making medication reconciliation clinically relevant and implementable [38]. Finally, the ideal length of time for performing medication reconciliation has yet to be defined and most likely depends on a number of additional factors such as number and/or type of medications a patient is taking as well as baseline patient characteristics (health literacy and comorbid conditions).

The implementation of health informatics tools for medication reconciliation could decrease demands on staff, standardize practice, reduce time and increase hospital compliance [40,41]. In fact, many organizations have begun developing and evaluating such systems as well as applications that link electronic medical records (EMR) and inpatient computerized physician order entry systems (CPOE) to facilitate effective medication reconciliation [41-44]. Such applications can generate an accurate pre-admission and discharge medication list, identify patients at risk of ADEs, allergies, adherence and persistence problems, as well as send alerts to patients, prescribers and other health care professionals [45,46]. Integration of e-health technology also offers the possibility of using administrative claims data repositories as a rich source of information. Several groups have evaluated electronic medication reconciliation forms [40,41,43,44] but the challenge of creating a clinical instrument that is both standard, in terms of content, and flexible, in terms of implementation at different settings and patient groups, remains. Based on direct and continuous clinical observation, we present a comprehensive list of clinically coherent tasks and subtasks, along with a detailed description of actions, grouped by type (admission or discharge) (Table 1). This list could provide additional insight for clarifying the roles and responsibilities of professionals and support the computerization of the process. Compilation and circulation of drug use information, currently acquired through *faxing* and *direct communication* with pharmacies, could be streamlined. Tasks, such as *documentation of medication changes*, could also be optimized electronically by the use of drop-down menus or other selection options that minimize the need to type free text. Time could then be appropriately re-allocated to validating the electronically populated community drug list and verifying *patient adherence* and *understanding*, tasks that require professional expertise and patient interaction.

In Quebec (Canada), the Medical Office of the 21st Century (MOXXI) [47], an integrated drug management system, provides near real-time information (within one hour) on dispensed prescriptions from community pharmacies through a secure virtual private network that links to the prescription claims system of the universal drug insurance program. A prospective randomized control trial is currently in place to determine if electronically enabled discharge reconciliation reduces the risk of adverse drug events, emergency room visits and readmissions 30 days post-discharge in Quebec [48]. It is expected that electronically enabled discharge reconciliation will improve adherence to medication reconciliation at discharge, the accuracy of the community-based drug history and effective communication of hospital-based treatment changes to community care providers. However, direct in-hospital observations are necessary to understand the specific requirements of the process in each hospital unit in order to refine existing or future electronic tools.

Three important study limitations are worth noting. First, we recognize the possibility of the Hawthorne effect [49] affecting this study, where workers may deliberately either over- or under-perform when they are being timed, depending on their attitudes towards the goal of the study. In order to minimize the Hawthorne effect, the research assistant held his or her laptop in a way that prevented the health professional being timed to view the screen and data sheet. A second limitation is the precision of the study, given the possibility of human timing errors. Although we used timing software to reduce such errors, occasionally it was not clear whether a task had been finished or not, in which case the research assistant had to ask the health care professional. In addition, our observations were limited to the hospitals’ regular operating hours and excluded evenings, nights and weekends, to characterize the medication reconciliation process during “optimal” staffing conditions. The medication reconciliation process may differ outside of regular hours of operation due to understaffing of health care professionals typically involved in mediation reconciliation (e.g. pharmacists) and/or presence of more junior staff with lesser direct supervision. In general surgery, medication reconciliation outside of regular hours may differ in terms of urgency, and/or acuity of cases. These represent unique challenges that warrant a separate study on medication reconciliation outside of regular operation hours. A third limitation is the sample size, which is small due to the high resource demands for conducting independent and continuous field observations. The strengths of this study lie in the fact that direct and continuous observation was used, which yielded a detailed description of the activities of all health care professionals and empirical evidence of the time devoted to each activity. Such information could be useful to accreditation agencies trying to standardize work in this field.

## Conclusion

More than half of ADEs occurring in hospitals result from medication discrepancies. While medication reconciliation is essential in reducing such ADEs and considered as a required hospital practice, challenges to its implementation result in poor hospital adherence. This study evaluated the number and type of different health care professionals involved and the time it took to complete tasks related to medication reconciliation at admission and discharge in three different hospital units. The results demonstrate that between one and three different health care professionals from two different disciplines, including medicine and pharmacy, were involved in the process at admission and/or discharge. There was considerable variation in terms of subtasks performed between and within units. Tasks independent of direct patient interaction, more specifically, reconciliation and documentation prior to and after meeting the patient, took more than twice the mean time required to complete tasks requiring direct patient interaction, such as discussion with patient. A better understanding of the admission reconciliation process in general surgery is needed. Standardization and the use of health informatics tools could potentially improve efficiency, reduce time-consumption and increase institutional ability to implement the process.

## Abbreviations

SD: Standard deviation; ADEs: Adverse drug events; MUHC: McGill University health centre; CPOE: Computerized provider order entry; EMR: Electronic medical records; MOXXI: Medical office of the 21st century.

## Competing interests

All the authors declare that they have no competing interests.

## Authors’ contributions

AM contributed to the conception and design of the study, interpretation of results, drafted and revised the manuscript. SK contributed to the interpretation of the results, drafted and revised the manuscript. KR contributed to the conception, design and analysis of the study. AH contributed to the conception and design of the study. RT contributed to the conception and design of the study and critically revised the manuscript. All authors read and approved the final manuscript.

## Pre-publication history

The pre-publication history for this paper can be accessed here:

http://www.biomedcentral.com/1472-6963/13/485/prepub
